# Visual Hallucinations following Coronary Artery Bypass Grafting: A Prospective Study

**DOI:** 10.3390/medicina58101466

**Published:** 2022-10-16

**Authors:** Marlene Tschernatsch, Jasmin El Shazly, Marius Butz, Sa-Ra Lie, Mesut Yeniguen, Tobias Braun, Georg Bachmann, Markus Schoenburg, Tibo Gerriets, Patrick Schramm, Martin Juenemann

**Affiliations:** 1 Heart and Brain Research Group, Kerckhoff Heart and Thorax Centre, Benekestrasse 2-8, 61231 Bad Nauheim, Germany; 2 Department of Neurology, Justus-Liebig-University, Klinikstrasse 33, 35385 Giessen, Germany; 3 Die Neurologen, Private Neurology Practice, Frankfurter Strasse 34, 61231 Bad Nauheim, Germany; 4 Department of Psychocardiology, Kerckhoff Heart and Thorax Centre, Benekestrasse 2-8, 61231 Bad Nauheim, Germany; 5 Department of General and Visceral Surgery, Gesundheitszentrum Wetterau, Chaumontplatz 1, 61231 Bad Nauheim, Germany; 6 Department of Radiology, Kerckhoff Heart and Thorax Centre, Benekestrasse 2-8, 61231 Bad Nauheim, Germany; 7 Department of Cardiac Surgery, Kerckhoff Heart and Thorax Centre, Benekestrasse 2-8, 61231 Bad Nauheim, Germany

**Keywords:** coronary artery bypass grafts, CABG, neurological testing, studies, neurology/neurologic, visual hallucinations

## Abstract

*Background and Objectives*: After major heart surgery, some patients report visual hallucinations that cannot be attributed to psychosis or delirium. This study aimed to investigate the hallucination incidence in patients after coronary artery bypass grafting with (on-pump) and without (off-pump) extracorporeal circulation. *Materials and Methods*: A total of 184 consecutive patients listed for elective on- or off-pump coronary artery bypass grafting were prospectively enrolled into the study. Preoperative baseline investigations 24–48 h before surgery (t0) and postoperative follow-up 24–48 h (t1) and 5–6 days (t2) after surgery included cognitive testing and a clinical visual acuity test (Landolt rings). Patients reporting visual hallucinations were interviewed using a structured survey to record the type, timing, duration, and frequency of their hallucinations. All the patients received a neurological examination and cranial magnetic resonance imaging if indicated. *Results*: Of the patients in the sample, 155 patients underwent on-pump bypass surgery, and 29 patients received off-pump surgery. Of these, 25 patients in the on-pump group, but none in the off-pump group, reported transient visual hallucinations (*p* = 0.020), which could not be attributed to stroke, delirium, psychosis, migraine, or severely impaired vision. Significant correlations were observed for the occurrence of visual hallucinations and the amount of nicotine consumption and aortic clamp/extracorporeal circulation time. *Conclusions*: Transient visual hallucinations occur in a noticeable proportion of patients after on-pump heart surgery. Knowledge of the phenomenon’s benignity is important for patients to prevent anxiety and uncertainty and for treating physicians to avoid unnecessary medication and drug-induced delirium.

## 1. Introduction

Neurologic and psychiatric consultants frequently assist patients who report visual hallucinations of coloured images of animate or inanimate objects after major heart surgery. The patients often distance themselves from this obviously false perception and show neither symptoms of delirium nor indications of psychosis; at best, they only seem to be concerned about the mere appearance of the hallucinations. A further difference is that a hallucination perceived in connection with delirium or psychosis is thought to be real by the patients, while a person affected by visual hallucinations without other neuropsychiatric symptoms is aware of the falsity of the perception. However, many also try to hide this experience for fear of being perceived as “mentally ill” or treated with medication for this reason. To distinguish such obviously false hallucinations from hallucinations in the context of complex clinical psychiatric conditions, the controversial term “pseudohallucinations” has been introduced [1,2].

Although the current literature provides further information on hallucinations mainly in the form of case reports, our previous case series shows that this clinically relevant phenomenon can occur in up to 11% of patients after coronary artery bypass grafting (CABG) [3,4,5,6]. A recently published investigation of a mixed cardiac surgery sample found an approximately 15% incidence of visual hallucinations in the first four postoperative days [7]. On the other hand, Eriksson et al. reported hallucinations in 58% of patients after CABG using extracorporeal circulation (ECC), regardless of the presence of delirium. At present, it is unclear whether the phenomenon is the direct result of cardiac surgery; in particular, the much-discussed connection with the ECC can at best be assumed, but not proven [8].

This study aimed to investigate the incidence of visual hallucinations in cardiosurgical bypass patients who underwent surgery with (on-pump) and without (off-pump) the use of ECC.

## 2. Materials and Methods

### 2.1. Patient Enrolment and Study Design

This single-centre prospective observational study was carried out at Kerckhoff Clinic, Bad Nauheim, Germany, and was approved by the Justus-Liebig University Giessen ethics committee (AZ 14/11, 19.04.2011). All patients provided written informed consent prior to participation.

Consecutive patients listed for elective on-pump and off-pump CABG were prospectively enrolled into this study. This research was planned as an explanatory study; therefore, no sample size calculation was carried out. We recruited adult patients 18 years or older who were scheduled for bypass surgery. Exclusion criteria were the inability to speak and understand German and pre-existing neurological or psychiatric disorders (in particular, cerebral infarction, traumatic brain injury with persistent deficits, manifest major depression, or dementia) that could interfere with neuropsychological testing and exploration regarding the occurrence of visual hallucinations. Each patient received standard intravenous narcotic anaesthetics, including sufentanil, remifentanil, propofol, and cisatracurium; none of the patients received ketamine. Postoperative pain was treated as per local protocol with metamizole and piritramide. All patients were admitted to the intensive care unit after surgery.

### 2.2. Study Process

As a cognitive screening test, the Mini Mental Status Examination (MMSE) was administered before and after surgery to exclude relevant preprocedural or postoperative cognitive decline as a risk factor for delirium. The preoperative baseline test 24–48 h before surgery (t0) included a detailed medical history survey (with particular regard to the presence of migraine) and a cognitive screening test assessing temporal and local orientation, memory, speech, language comprehension, and motor skills using the MMSE [9], as well as a clinical visual acuity test (Landolt rings) [10]. Alcohol consumption, history of drug abuse, and nicotine consumption (packs smoked daily × years of smoking = pack-years) were surveyed in a structured interview.

The following sociodemographic data and intra- and postoperative parameters were recorded: age, sex, body mass index, American Society of Anaesthesiologists (ASA) classification, alcohol and nicotine consumption, type and duration of surgery, ECC time, aortic clamping time, minimum temperature, ventilation time, and length of stay in the intensive care unit.

The MMSE was administered again 24–48 h (t1) and 5–6 days (t2) after surgery, and the patients were specifically asked about the occurrence of visual hallucinations. If visual hallucinations occurred during this time, the patients were interviewed in detail with a structured survey recording the type of hallucination, the timing, the duration, and the frequency. Patients were also asked to sketch the visual phenomena on a piece of paper. The occurrence of delirious symptomatology was assessed by the medical and nursing staff. In the event of the suspicion of delirious symptomatology, patients were again examined by a neurologist.

### 2.3. Magnetic Resonance Imaging (MRI)

There is an increased risk for so-called silent brain infarctions associated with CABG operations, many of which display no or unrecognized clinical signs [11]. To exclude a silent brain infarction in the visual system causing the pseudohallucinations, a cranial MRI was performed with a 1.5 T device (SONATA; Siemens, Erlangen, Germany). The imaging protocol consisted of a proton- and T2-weighted turbo spin-echo sequence, a T1-weighted TSE, a T2-weighted turbo-inversion recovery sequence, and a diffusion-weighted echo-planar imaging sequence. Apparent diffusion coefficients (ADCs) were calculated for each pixel, correlated with a grey scale, and finally composed to ADC maps. Two experienced observers (T.G. and G.B.) evaluated the MRI scans independently. The postoperative diffusion-weighted sequence was used for the registration of acute ischemic lesions.

### 2.4. Statistical Analyses

We tested the assumption of normality using the Shapiro–Wilk test and the homogeneity of variance using the Levene test. To identify potential risk factors for postoperative visual hallucinations, we conducted bivariate analysis of demographic data, relevant medical data, intraoperative data, and postoperative clinical variables. The frequencies were compared using the chi-squared test or Fisher’s exact test, and the means of normally distributed data were compared using Student’s *t*-test. Continuous variables that did not meet the criteria of the parametric test were evaluated by the Mann–Whitney U test. The incidence of visual hallucinations and all pre-, intra-, and postoperative categorical parameters are given as frequency and percentage. The means and standard deviation are given for all metric variables. Correlations were investigated using Pearson’s correlation in cases of metric variables or Spearman’s correlation in cases of variables that were not normally distributed or ordinal variables. To investigate a possible association between the MMSE score and the occurrence of a visual hallucination, repeated ANOVA was calculated with groups as the between-subject factor (occurrence of visual hallucinations vs. no visual hallucinations) and assessment time as the within-subject factor (baseline, t1, and t2). The criterion for statistical significance was set at *p* < 0.05. For statistical analysis, we used the Statistical Package for Social Science software (SPSS 25; IBM^®^, Chicago, IL, USA).

## 3. Results

### 3.1. Study Population

Over a period of 4 years, 179 patients undergoing elective bypass surgery with the use of ECC gave informed consent for participation. Of these, 24 patients were not included in the data analysis because an exclusion criterion (e.g., postoperative delirium) was discovered after the informed consent procedure, the operation was cancelled, or the patient withdrew consent. Other patients could not be interviewed due to organizational reasons or postoperative complications. Therefore, 155 patients undergoing bypass surgery with the use of ECC (on-pump) were included in the analysis. In addition, 29 patients enrolled in the study underwent bypass surgery without the use of ECC (off-pump), all of whom were included in the data analysis.

Of the 155 patients undergoing on-pump surgery, a total of 25 (16.1%) reported pseudohallucinations at t1, t2, or both, whereas of the 29 patients that underwent off-pump surgery, none (0%) had pseudohallucinations (*p* = 0.20).

The baseline and clinical characteristics of the on- and off-pump patients are shown in Table 1.

A total of 184 patients were included in the data analysis (155 patients on-pump, 29 patients off-pump). The two groups differed significantly regarding the alcohol consumption variable: more patients in the off-pump group stated they consumed alcohol on a regular basis (*p* = 0.007, see Table 1). None of the patients reported a history of drug abuse. Regarding the medical history and the indication for surgery, differences were noted in the ejection fraction, with a higher ejection fraction in the off-pump group (*p* = 0.013), and in the severity of the cardiovascular disease, with more patients in the off-pump group suffering from one-vessel disease and fewer from three-vessel disease (*p* = 0.004). With respect to the intraoperative data, the duration of surgery (longer duration of surgery in the on-pump group, *p* < 0.001), the lowest body temperature (lower body temperature in the on-pump group, *p* < 0.001), and the duration of ventilation (longer ventilation times in the on-pump group, *p* = 0.026) differed significantly between the groups (see Table 1).

### 3.2. Prevalence of Visual Hallucinations

Of the 155 on-pump patients, 25 patients reported visual hallucinations (16.1%, six females, 19 males; age 67.32 ± 7.59 years), whereas in the off-pump group, none of the patients described this experience (*p* = 0.020). None of the patients reported any other form of hallucination or delusion (for example, auditory hallucinations) or the occurrence of hallucinations in the past. The visual hallucinations could not be attributed to stroke, delirium, psychosis, migraine, or severely impaired vision.

Subsequently, bivariate correlations were calculated between the occurrence of visual hallucinations and the pre-, intra-, and postoperative variables. There was a positive correlation between the ECC time (*r* = 0.146, *p* = 0.048) and aortic clamp time (*r* = 0.175, *p* = 0.018) with the occurrence of pseudohallucinations. Furthermore, a significant correlation for nicotine consumption (number of pack-years, *r* = 0.180, *p* = 0.015) was observed.

In addition, on-pump patients reporting visual hallucinations were compared with on-pump patients reporting no visual hallucinations. The groups differed significantly regarding the pack-years variable (*p* = 0.034), with a higher value of pack-years for the patients with visual hallucinations.

In all other pre-, intra-, and postoperative variables, no group differences could be noted (see Table 2).

### 3.3. Clinical and MRI Findings

None of the patients who reported visual hallucinations suffered from visual impairment or migraine. In the absence of focal neurological deficits, the detailed neurological examinations revealed neither neurological nor psychiatric pre-existing conditions. None of the patients showed acute changes or fluctuations in mental state, inattention, signs of disorganized thinking, or an altered state of consciousness, which are necessary for the diagnosis of delirium. No significant interaction effect was observed between the MMSE score and the occurrence of visual hallucinations. An MRI of the neurocranium was performed in nine patients and a computed tomography of the neurocranium in one patient with visual hallucinations; an acute ischemic brain lesion was detected in three patients. The lesions were supratentorial cortical/subcortical microinfarcts (3–6 mm) within the territory of the middle cerebral artery. The lesions did not affect the visual pathway and were clinically silent.

### 3.4. Phenomenology of Visual Hallucinations

**Figure 1:**
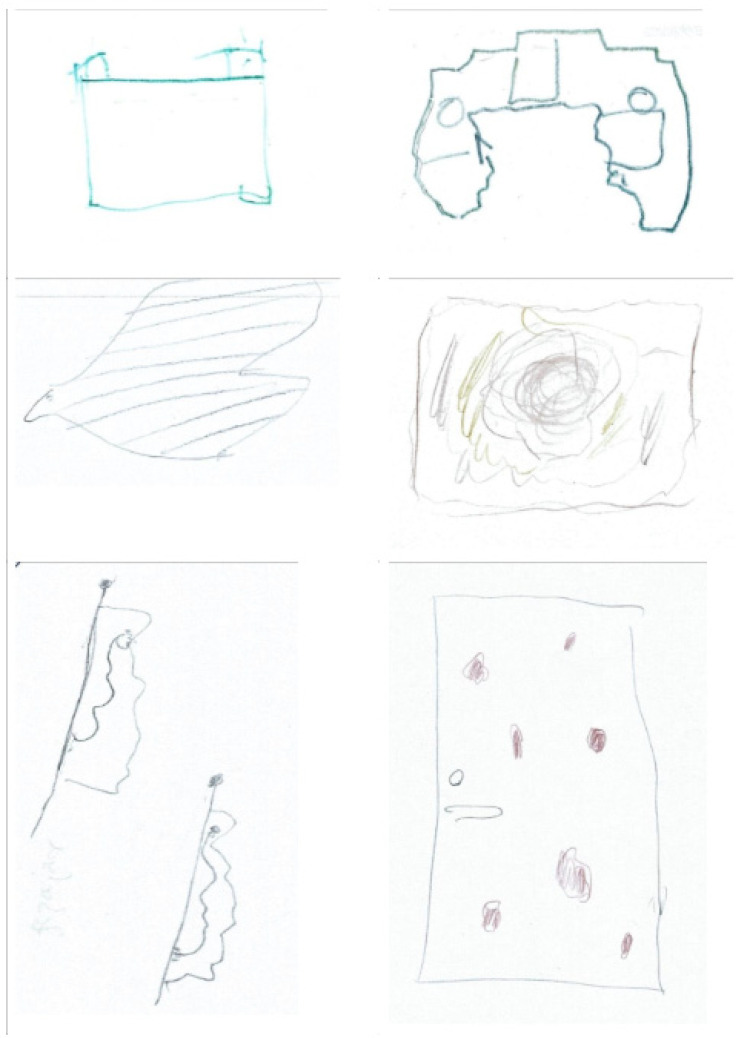
Selected illustrations of the visual hallucinations drawn by the patients.

In the structured interviews, 22 of 25 patients reported having seen inanimate objects such as geometrical figures, dots, spots, lightning, and plants, 15 described moving pictures, and 6 reported animate objects such as animals or pictures with moving content. Nineteen patients hallucinated in colour, and fifteen reported moving objects. The appearance of the visual phenomena was not related to the time of day, the patients’ activity, or the light conditions. Twenty-three patients affirmed that the images only occurred while their eyes were open, and two patients reported that the images only occurred while their eyes were closed. Two patients stated that the hallucinations scared them, but all twenty-five patients were able to distance themselves completely from the hallucinations, regarding them as an unreal phenomenon. Twelve patients had only one hallucination, and ten patients reported up to five hallucinations. The phenomena were usually seen for a few seconds to a few minutes but lasted longer than 5 min for six patients. For 16 patients, the symptoms appeared within the first 48 h after surgery, and for the others, it was not until the fifth day. The optical hallucinations were often perceived on blank surfaces such as the floor, walls, or doors. Selected illustrations of the visual hallucinations are shown in Figure 1.

## 4. Discussion

This study aimed to investigate the occurrence of visual hallucinations after bypass surgery. In a sample of 184 consecutive patients, 155 underwent on-pump bypass surgery with extracorporeal circulation, whereas 29 underwent off-pump surgery. Twenty-five patients (16%) in the on-pump group and no patients in the off-pump group reported transient visual hallucinations (*p* = 0.020). The hallucinations mostly occurred within the first 2 days after the operation; they usually involved inanimate coloured objects and were perceived as unreal phenomena in the sense of pseudohallucinations.

The magnitude of the incidence was estimated in our group’s earlier published case series of 100 non-delirious patients, of whom 11 reported visual hallucinations after CABG or aortic valve replacement with the use of ECC [3]. The type and duration of the described hallucinations appear to be comparable. Recently, Ottens et al. reported an incidence of hallucinations of 21.9% after cardiac surgeries such as CABG, single and multiple valve replacement, thoracic aortic surgery, and combined procedures. Among the hallucinations of different modalities, 70.5% were visual, and only 4.0% of patients reported anxiety or discomfort associated with the hallucinations. Although an association existed between postoperative hallucinations and delirium in the study, most patients with hallucinations did not experience delirium, and independent predictors for postoperative hallucinations could not be identified [7]. Eriksson and colleagues investigated the occurrence of delirium after CABG surgery and reported that, in their sample, more than 50% of patients complained of hallucinations regardless of whether they suffered delirium [8]. In comparison, our studies on non-delirious patients showed a significantly lower incidence.

In our study, a validated detection tool such as the Questionnaire for Psychotic Experiences (QPE) to detect hallucinations was not used, since recruitment of patients took place before it was published, which may partly explain the relatively lower incidence [12]. MMSE alone may not be suitable for the detection of delirium with a high sensitivity. However, patients with high MMSE scores are unlikely to develop delirium, as has been shown by Kiely et al., who found a correlation between the development of delirium and the MMSE score: patients who developed delirium had an average score of 12.6, subsyndromal patients scored 16.9–21.5, while patients that did not develop delirium had a score of 25.7 ± 3.2. Since all patients in our study had an MMSE score >28, it is quite unlikely that they developed delirium, especially because the postoperative progress was uncomplicated without any delay [13].

While there was no correlation with whether patients ever smoked in the past or present, bivariate analysis indicated a positive correlation between the occurrence of visual hallucinations and higher pre-existing nicotine use (number of pack-years). In population-based surveys, psychotic symptoms and delusion-like experiences are more common among daily tobacco smokers [14,15]. However, the visual hallucinations described in the present study should not be evaluated in the context of a psychotic episode because patients were clearly distanced from the hallucinations and other symptoms were absent. In addition, a connection with withdrawal symptomatology is difficult to establish because some of the affected patients quit smoking a long time ago.

Another disease of differential diagnostic importance is Charles–Bonnet syndrome, which is predominantly observed in older patients suffering significantly impaired vision, for example, due to macular degeneration, retinopathy, or corneal disease [16,17]. Psychologically/psychiatrically healthy patients with Charles–Bonnet syndrome often report lively and artistic hallucinations from which they clearly distance themselves but which can frighten them. In addition to intraocular pathologies, damage to all parts of the visual tract can lead to this symptomatology. It has been discussed whether these hallucinations are expressions of a “release phenomenon” caused by the deafferentiation of the visual input in the extrastriatal visual associative cortex [1,18,19,20]. Therefore, in the case of visual hallucinations, an MRI of the neurocranium was used in the present study to evaluate the presence of a structural lesion of the brain, especially along the visual tract. In addition, repeated tests of visual acuity and assessment of the visual field were performed as part of the neurological examination so that corresponding changes in vision could be reliably detected and documented. The MRI revealed no structural changes in the visual tract, nor did patients with pseudohallucinations show a significantly lower visual acuity; hence, attribution of the hallucinations to this syndrome is difficult.

The different types of hallucinations can be mapped to the corresponding cortical area using data derived from electrical brain stimulation [21]. The visual hallucinations can be related to the occipital and temporal cortical area (striate cortex, peristriate cortex, superior occipital cortex, temporo-occipital junction, and inferior temporal gyrus), which are all supplied through the posterior cerebral artery. Six of the twenty-five patients with visual hallucinations described additional hallucinations of different sensory modalities, such as hearing louder or numbness of extremities; these can be mapped to the temporal and insular cortex, which are supplied by the posterior and middle cerebral artery, respectively. Microemboli after ECC are distributed in various brain regions, which may be a hint at a distinct clinical correlation, even though it is unrecognisable in an MRI scan [22,23].

It seems almost undisputed that the use of ECC increases the risk of cerebral microemboli. However, the extent to which microemboli can contribute to cerebral complications such as brain infarctions and postoperative cognitive deficits is still debated. In our sample, the occurrence of visual pseudohallucinations correlated not only with the use of ECC but also its duration and the aortic clamp time. It has been shown that the quantity of microemboli increases with the length of the ECC; thus, the correlation found here between hallucination and length of ECC supports the hypothesis of a microembolic causation [24]. However, postoperative neurological deficits as a symptom of a cerebral infarction could not be objectified, and the image-based detection of acute cerebral ischemia by means of diffusion-weighted imaging only showed microinfarcts whose size and location did not explain visual hallucinations.

Multimodal MRI studies investigating on- and off-pump bypass surgery showed not only an increased number of cerebral microembolisms in the case of ECC surgery, but also that the majority of them can be visualized in brain areas supplied by the vertebrobasilar system [25]. Therefore, it is conceivable that visual hallucinations are the consequence of microembolic damage to the visual pathway that might be below the detection threshold of MRI. In this context, the association of visual hallucinations with extensive nicotine use could possibly indicate chronic cerebrovascular pre-damage resulting in a reduced vascular compensatory capacity at times of or in regions with hypoperfusion. After the damage has occurred, a neurotransmitter imbalance (and thus, hyperexcitability of certain neurons) may occur during a cortical recovery process [26]. Consequently, visual hallucinations could be seen as the clinical correlate of this release phenomenon.

A limitation of the present study is certainly the considerably different group size. The recruitment process took longer than estimated, for the exclusion criteria were rigidly applied, and the number of off-pump surgeries decreased during the recruitment interval. This was also happening throughout Germany, with a decreasing proportion of off-pump procedures [27]. This resulted in a relatively small off-pump group; however, since the recruitment interval took longer than estimated, we decided to work with the smaller off-pump group to avoid extending the study even further. However, even with a small sample size, the difference between the groups was significant, with zero off-pump patients having hallucinations.

Nevertheless, relative to the large number of operations performed with ECC, patients tend to underreport visual hallucinations of their own accord. Patients should be specifically asked about such symptoms so that they can be informed about the benignity of pseudohallucinations to prevent the possible development of anxiety and insecurity. The physicians in charge should also be informed about the symptoms to prevent unnecessary medication and drug-provoked delirium, which have negative consequences on mortality and morbidity [28]. This should be considered especially in patients with high nicotine consumption in the past with a high number of pack-years and a longer aortic clamp and extracorporeal circulation time.

## 5. Conclusions

In conclusion, transient visual pseudohallucinations occur in a noticeable proportion of patients after on-pump heart surgery. Knowledge of the phenomenon’s benignity is important for patients to prevent anxiety and uncertainty and for treating physicians to avoid unnecessary medication and drug-induced delirium.

## Figures and Tables

**Table 1:** Comparison of preoperative, intraoperative, and postoperative data of the on-pump vs. off-pump groups.

Variables	On-Pump	Off-Pump	*p*-Value
	(*n* = 155)	(*n* = 29)	
Visual hallucinations (total)	25 (16.1%)	0 (0%)	0.020 *
Patient characteristics			
Sex			0.07
female	33 (21.3%)	2 (6.9%)	
male	122 (78.7%)	27 (93.1%)	
Age (years)	67.71 (9.27)	68.03 (9.93)	0.705
Body mass index (kg/m^2^)	28.34 (4.22)	27.71 (4.72)	0.296
Alcohol and nicotine use			
Nicotine use			0.089
current	36 (23.2%)	2 (6.9%)	
never	55 (35.5%)	15 (51.7%)	
former	64 (41.3%)	12 (41.4%)	
Pack-years	17.55 (20.38)	10.17 (12.92)	0.067
Alcohol consumption			0.007 *
none	75 (48.4%)	12 (41.4%)	
occasional	65 (41.9%)	8 (27.9%)	
regular	15 (9.7%)	9 (31.0%)	
Medical history			
Ejection fraction (%)	58.62 (9.43)	62.66 (6.80)	0.013 *
History of myocardial infarction	49 (31.6%)	10 (34.5%)	0.761
Medical history of migraine	5 (3.2%)	1 (3.4%)	0.955
ASA classification			0.322
1	4 (2.6%)	1 (3.4%)	
2	144 (93.5%)	25 (86.2%)	
3	6 (3.9%)	3 (10.3%)	
Coronary artery disease			0.004 *
1-vessel	1 (0.6%)	3 (10.3%)	
2-vessel	16 (10.3%)	3 (10.3%)	
3-vessel	138 (89.0%)	23 (79.3%)	
Intraoperative data			
Surgery time (min)	219.67 (46.42)	176.41 (48.91)	<0.001 *
Low point during hypothermia (°C)	35.08 (0.85)	35.66 (0.59)	<0.001 *
Ventilation duration (min)	621.38 (219.37)	516.45 (269.31)	0.026 *
Time in intensive care unit (h)	35.54 (24.48)	28.55 (12.08)	0.116
Baseline data			
Vision left	0.80 (0.24)	0.87 (0.19)	0.167
Vision right	0.84 (0.21)	0.85 (0.19)	0.921
MMSE t0	28.35 (1.53)	28.59 (1.15)	0.667
Follow-up examinations			
MMSE t1	27.40 (2.55)	27.55 (2.0)	0.831
MMSE t2	28.34 (1.73)	28.93 (1.44)	0.063

Note: All categorical variables are reported as frequency and percentage. The metric variables are reported as mean and standard deviation. Values are mean ± SD or number (%). * Significant differences with *p* < 0.05. ASA = American Society of Anaesthesiologists; MMSE = Mini Mental Status Examination

**Table 2:** Comparison of preoperative, intraoperative, and postoperative data of the patients with visual hallucinations vs. without visual hallucinations.

Variables	Visual Hallucinations	No Visual Hallucinations	*p*-Value
	(*n* = 25)	(*n* = 130)	
Patient characteristics			
Sex			0.718
female	6 (24.0%)	27(20.8%)	
male	19 (76.0%)	103 (79.2%)	
Age (years)	67.32 (7.59)	67.78 (9.58)	0.647
Body mass index (kg/m^2^)	27.95 (3.35)	28.42 (4.37)	0.688
Alcohol and nicotine use			
Nicotine use			0.296
current	4 (16.0%)	51(39.2%)	
never	8 (32.0%)	28 (21.5%)	
former	13 (52.0%)	51 (39.2%)	
Pack-years	23.46 (18.78)	16.44 (20.55)	0.034 *
Alcohol consumption			0.082
none	9 (36%)	66 (50.8%)	
Occasional	14 (56%)	51 (39.2%)	
regular	2 (8.0%)	13 (10%)	
Medical history			
Ejection fraction (%)	56.48 (10.33)	59.03 (9.23)	0.189
Myocardial infarction	10 (40%)	39 (30%)	0.325
Migraine	0	5 (3.8%)	0.329
ASA classification			0.094
1	2 (8%)	2 (1.5%)	
2	23 (92%)	122 (93.8%)	
3	0 (0%)	6 (4.6%)	
Coronary artery disease			0.47
1-vessel	0	1 (0.8%)	
2-vessel	1 (4%)	15 (11.5%)	
3-vessel	24 (96%)	114 (87.7%)	
Intraoperative data			
Surgery time (min)	225.88 (49.20)	218.48 (45.98)	0.481
Extracorporeal circulation time (min)	92.48 (24.05)	90.91 (29.64)	0.541
Aortic clamp time (min)	63.80 (20.72)	58.68 (20.43)	0.308
Low point during hypothermia (°C)	35.01 (1.12)	35.09 (0.79)	0.833
Ventilation duration (min)	615.32 (207.36)	622.55 (222.35)	0.832
Time in the intensive care unit (h)	38.84 (28.48)	34.91 (23.71)	0.728
Baseline data			
Vision left	0.81 (0.22)	0.80 (0.25)	0.953
Vision right	0.80 (0.29)	0.85 (0.19)	0.819
MMSE t0	28.68 (1.46)	28.29 (1.54)	0.16
Follow-up examinations			
MMSE t1	27.52 (3.37)	27.38 (2.37)	0.199
MMSE t2	28.44 (1.96)	28.32 (1.69)	0.416

Note: All categorical variables are reported as frequency and percent. The metric variables are reported as mean and standard deviation. Values are mean ± SD or number (%). * Significant differences with *p* < 0.05.

## Data Availability

Not applicable.
